# Exploring the antigenic response to multiplexed immunizations in a chicken model of antibody production

**DOI:** 10.1016/j.heliyon.2017.e00267

**Published:** 2017-03-16

**Authors:** Tina M. Kousted, Otto Kalliokoski, Sofie K. Christensen, Jakob R. Winther, Jann Hau

**Affiliations:** a Department of Experimental Medicine, University of Copenhagen, Denmark; b Linderstrøm-Lang Centre for Protein Science, Department of Biology, University of Copenhagen, Denmark

**Keywords:** Biotechnology, Immunology

## Abstract

Hens have a tremendous capacity for producing polyclonal antibodies that can subsequently be isolated in high concentrations from their eggs. An approach for further maximizing their potential is to produce multiple antisera in the same individual through multiplexed (multiple simultaneous) immunizations. An unknown with this approach is how many immunogens a single bird is capable of mounting a sizeable antigenic response toward. At what point does it become counter-productive to add more immunogens to the same immunization regimen?

In the present study we were able to demonstrate that the competing effects of co-administering multiple immunogens effectively limit the antibody specificities that can be raised in a single individual to a fairly low number. Two potent model immunogens, KLH and CRM_197_, were administered together with competing antigens in various concentrations and complexities. With an upper limit of 1 mg protein material recommended for chicken immunizations, we found that the maximum number of immunogens that can be reliably used is most likely in the low double digits.

The limiting factor for a response to an immunogen could not be related to the number of splenic plasma cells producing antibodies against it. When administering KLH alone, up to 70% of the IgY-producing splenic plasma cells were occupied with producing anti-KLH antibodies; but when simultaneously being exposed to a plethora of other antigens, a response of a comparable magnitude could be mounted with a splenic plasma cell involvement of less than 5%.

Two breeds of egg-layers were compared with respect to antibody production in an initial experiment, but differences in antibody productivity were negligible.

Although our findings support the use of multiplexed immunizations in the hen, we find that the number of immunogens cannot be stretched much higher than the handful that has been used in mammalian models to date.

## 1 Introduction

Polyclonal antibody production is a less sophisticated technology than is monoclonal antibody production; yet, polyclonal antibodies make up a substantial share of the total yearly sales in the antibody market. Polyclonals offer advantages in the production stages: the antibodies can be produced simply, cheaply, and with a short turnover time from purification of an immunogen to the isolation of an antiserum. Traditionally, polyclonals are produced in mammalian species – rabbits being the most utilized species – but a strong case has been made for using birds, specifically hens [1,2,3,4]. The principal advantage is that antibodies are deposited in the hens’ eggs through maternal transfer [5,6]. The antibodies are then, with relative ease and a high degree of purity, isolated from egg yolks [7,8]. The breeds used in modern egg-laying facilities will lay an egg nearly every day [9] for a year from when the hen is 4–6 months old [5,10], and healthy hens have been known to lay eggs well-past their tenth year [11]. With the egg yolk containing antibodies at concentrations similar to those in the hen’s serum, a hen will in one month produce amounts of antibodies similar to those obtained through blood samplings from a rabbit in an entire year [12,13].

Since the pioneering work of isolating chicken IgY (also sometimes referred to as avian IgG) from eggs [14,15,16], a number of commercial companies have established themselves on the polyclonal antibody market, offering chicken antibodies for research and diagnostic use; however, their market share is limited. A possible reason contributing to these companies’ lack of market penetration is their inability to effectively utilize the capacity offered by the chicken in antibody production. One approach for better utilizing this tremendous capacity is by producing antiserum with multiple specificities in the same animal: Isolation and purification methods for antibodies are easily scalable whereas housing and husbandry of animals are not. By simultaneously producing multiple antisera in the same animal, production costs and animal numbers can be reduced manifold [17,18].

Producing multivalent antiserum in an animal requires a solid understanding of how the immune system responds to multiple simultaneous immunizations. Immunizations with multiple immunogens (multiplexed immunizations) have been used to great effect in mammalian species [18,19,20,21] – of particular note is the use of multiplexed immunizations in generating the Human Protein Atlas [22,23,24] – and to a limited extent been explored in chickens [25,26,27]. Many of the fundamental concepts are still poorly understood [28,29,30], however, and all but unknown for birds. In the present study we set out to explore a fundamental concept: to determine the number of antigens a single individual (bird) can respond to.

Working with a limited injection volume [2,31], it stands to reason that there must be an upper limit to the number of immunogens that can be combined in one immunization. In controlled immunizations more than ten immunogens have rarely been used (e.g. [20]). But immunizations using whole, or fractionated, serum have been long-used to generate antibodies against major serum proteins [32,33], suggesting that the capacity for producing useful polyclonal serum is much higher. Consequently we hypothesized that the hen’s immune system could be used to generate polyclonal antiserum toward thousands of immunogens simultaneously.

Chickens lack lymph nodes and the Bursa of Fabricius – the organ responsible for B-cell development in birds – involutes and appears to have little to no function in adult birds [34]; consequently the spleen plays a central role in the mature hen’s adaptive immune response [35]. If the capacity of the spleen to host plasma cells for producing circulating antibodies is limited, and if a given number of plasma cells is required for an adequate antigenic response, estimating the sizes of these populations should offer a way of predictably estimating the maximum number of antigens an individual can produce high-avidity antiserum against. To test our hypothesis we designed experiments to identify the critical amount of a model immunogen required to elicit a significant antigenic response, when administered in the presence of a complex mixture of competing antigens. In addition, we set out to quantify the number of splenic plasma cells associated with these responses.

Producing a matrix of competing antigens from commercially available purified proteins would make for a prohibitively expensive study design given that we expected to need thousands of unique species. Instead we opted for a reversed design, starting with a natural source offering a complex composition of proteins, and using chromatographic techniques to derive less complex matrices from this source. In addition, we chose to investigate whether the antigenic response differed greatly between two common breeds of laying hens.

## 2 Material and methods

### 2.1 Experiments

#### 2.1.1 Experiment 1: comparing the antigenic response in two chicken breeds

To determine whether there are large discrepancies between chicken breeds in relation to antigenic response, we compared the response of two of the most common egg-laying breeds utilized at farms in Denmark: ISA Warren (n = 14) and Lohmann LSL (n = 14). On day 0, when chickens were 21 weeks of age (4–6 weeks after any previous vaccinations), the hens were randomly allotted to receive a subcutaneous immunization with KLH (Keyhole Limpet Hemocyanin) of up to 100 μg (Table 1). Blood samples were drawn immediately prior to booster immunizations, which were given on days 14 and 28. A final blood sample was drawn on day 33 before the experiment was terminated. Concentrations of IgY antibodies against KLH (α-KLH IgY) and total circulating concentrations of IgY were determined in the blood samples (EDTA plasma). When the experiment was terminated, spleens were harvested from the Lohmann LSL hens receiving the highest dose (to act as a reference in Experiment 3).

#### 2.1.2 Experiment 2: titration of KLH and diphtheria toxin against a mixture of competing antigens.

To determine the concentration at which an immunogen is able to elicit a measurable antigenic response in the presence of a complex matrix of (competing) proteins, Lohmann LSL hens (n = 50) were immunized with KLH, diphtheria toxin and protein mixes of differing complexity (Table 2). The hens received their first immunization (Day 0) at 14 weeks of age (12 weeks after any previous vaccination) and were booster immunized on day 26. Blood samples (serum) were drawn immediately prior to the boosting and on day 39. IgY and α-KLH IgY concentrations were determined in the blood samples (serum).

#### 2.1.3 Experiment 3: titration of competing antigens against a constant dose of KLH

To determine the effect of a complex matrix of competing proteins on the antigenic response toward a given immunogen, Lohmann LSL hens (n = 26) were immunized with KLH and a protein mixture (Table 3). The hens received their first immunization (Day 0) at 18 weeks of age (5 weeks after any previous vaccination). Blood samples were drawn immediately prior to booster immunizations, which were given on days 14 and 28. A final blood sample was drawn and spleens were harvested on day 33. IgY/α-KLH IgY concentrations were determined in the blood samples (EDTA plasma). Splenocytes were analyzed for cells producing IgY and α-KLH IgY.

### 2.2 Housing and husbandry

ISA Warren hens were purchased from Bangs Hønniker Aps (Hoejtoftegaard, Denmark) and Lohmann LSL hens were purchased from Ova Production AB (Vittinge, Sweden; Experiment 2) or Triova Aps (Herlufmagle, Denmark; Experiment 3). Chickens involved in Experiments 1 and 3 were housed in groups of up to 15 individuals (one rooster per cage). Pens, measuring 5.76 m^2^ (2.4 × 2.4 m, height 1.8 m), were lined with wood shavings and equipped with perches, nests and a dust bath (sand and soil). Water and feed – a mixture of grains, corn and pelleted diet (DLG a.m.b.a, Copenhagen, Denmark) – was supplied *ad libitum* and food enrichment items (e.g. fruits, vegetables, seeds and mealworms) were supplied daily. Room temperature was maintained at approximately 18 °C and lights were controlled to give 14 hours of artificial daylight. Chickens involved in Experiment 2 were housed in groups of 12–13 individuals (one rooster per cage). Wire cages, measuring 2 m^2^ (2 × 1 m, height 0.55 m), were equipped with perches, nests and a dust bath (finely crushed stone and wood shavings). Water and feed – a mixture of grains, mussel shells and pelleted diet (AB Johan Hansson, Uppsala, Sweden) – were supplied *ad libitum*. Room temperature was maintained at 18–22 °C and lights were controlled to give 15 hours of artificial daylight.

#### 2.2.1 Procedures

For immunizations, the hens were restrained and a total of 400 μl of a 1:1 stable immunogen/FIA (Freund’s incomplete adjuvant; Statens Serum Institut, Copenhagen, Denmark) emulsion was deposited subcutaneously in three injection sites on the chest. Blood samples (approximately 2 ml) were drawn from the wing vein of the hens into EDTA-treated vials (Experiments 1 and 3) or allowed to coagulate in non-EDTA vials (Experiment 2). Blood samples were centrifuged at 5000 g for 10 minutes and samples were decanted and stored at −20 °C until analysis. The hens were euthanized by decapitation and spleens were dissected and immediately placed into a cell culture medium.

#### 2.2.2 Ethics statement

Experiments 1 and 3 were performed in AAALAC (Association for Assessment and Accreditation of Laboratory Animal Care) International accredited facilities under the supervision of a local ethics committee. All procedures were approved by the Animal Experiments Inspectorate under the Danish Ministry of Food, Agriculture and Fisheries (license number 2012-15-2934-00077). Experiment 2 was performed at Ova Production AB (Vittinge, Sweden) where all procedures were approved by the regional Ethics Committee.

### 2.3 Antigens

Model immunogens, KLH (Sigma Aldrich, St. Louis MO, USA; cat. no. 70557) and lyophilized diphtheria toxin variant CRM_197_ (Merck Millipore, Billerica MA, USA; cat. no. 322327), were obtained from commercial vendors. Protein mixtures of differing complexities, but comparable concentrations, were prepared by isolating water soluble proteins from human placental tissue, chromatographical fractionation, and pooling of select fractions.

#### 2.3.1 Preparing a water-soluble protein starting material

Pooled and rinsed human placental tissue was processed into a course homogenate, passed through a coarse sieve, and centrifuged to remove particulates (3 × 30 minutes at 20,000 g, 4 °C). The final supernatant was filtered, and stored at −20 °C with 0.1 mM phenylmethylsulfonyl fluoride. The crude placental extract was then processed to obtain a water-soluble protein fraction: The thawed material was diluted 10-fold in a Tris buffer (20 mM Tris-HCl pH 7.4 with 150 mM NaCl), ammonium sulphate was added (a saturated solution was added dropwise to obtain a 50% solution) and the suspension was left to precipitate overnight at 4 °C. After centrifugation (20,000 g, 30 minutes, 4 °C) the pellet was resuspended in Tris buffer; insoluble proteins were separated by centrifugation and discarded.

#### 2.3.2 Fractionation by size exclusion chromatography

The placental protein solution was fractionated batch-wise using size exclusion chromatography on a HiLoad 26/60 Superdex200 pg column (GE Healthcare, Chicago IL, USA) equilibrated in Tris buffer (cleaned between runs with 0.5 M NaOH). Placental protein (100 mg, estimated by spectrophotometry at 280 nm, in 4 ml) was loaded, and 4 ml fractions were collected at flow rate 2.5 ml/min. Elution profiles (recorded at 280 nm) were aligned and corresponding fractions were pooled across batches to form equal volume eluates. The pooled eluates were concentrated using Amicon Ultra-15 centrifugal filters (Merck Millipore, Billerica MA, USA) and combined to form three protein mixtures with comparable amounts of total protein but differing levels of complexity.

#### 2.3.3 SDS-PAGE

Pooled eluates and protein mixtures were separated and imaged by SDS-PAGE. Samples were reduced in with 100 mM 1,4-Dithiothreitol in a loading buffer (10% Glycerol, 50 mM Tris-HCl pH 6.8, 2% SDS, 0.01% Bromophenol blue), boiled for 2 minutes and loaded onto a freshly cast 11% polyacrylamide gel. Electrophoresis was performed in a standard Tris-glycine buffer system at 70 V and 150 V to allow migration of proteins through the stacking and separation gel, respectively. The gels were stained with Coomassie Blue.

### 2.4 Enzyme-linked immunosorbent assays (ELISAs)

#### 2.4.1 Sandwich ELISA for quantification of total IgY

Clear 96-well plates (Costar high binding; Corning Inc., Corning NY, USA) were incubated (1 hour at 37 °C) with 100 μl/well 0.5 μg/ml goat anti-chicken IgY (Southern Biotech, Birmingham AL, USA; cat. no. 6100-01). The plates were washed in PBS (pH 7.4, 0.05% Tween 20), blocked (1 hour at 37 °C) with 200 μl/well PBS with 1% BSA and washed again. Chicken IgY standards (Merck-Millipore; cat. no. AC146) and serum/plasma samples were diluted in PBS (0.1% BSA) and analyzed in duplicate (100 μl/well, incubated 1 h at 37 °C). The plates were washed and 100 μl/well horseradish peroxidase (HRP) conjugated rabbit anti-chicken IgY (Sigma Aldrich; cat. no. A9046), diluted 1:50,000 in PBS (0.1% BSA), was added. Following incubation (1 hour at 37 °C), the plates were washed before addition of 50 μl/well TMB (3,3′, 5,5′-tetramethylbenzidine, Sigma Aldrich; cat. no. T0440). The plates were developed for 30–40 minutes in the dark before the reaction was terminated by adding 100 μl/well 1 M H_2_SO_4_ and absorbances were measured at 450 nm (Multiskan EX; Thermo Fisher Scientific, Waltham MA, USA). The assay showed excellent parallelism and an average spike recovery of 100% and 92% for serum and plasma samples, respectively (spikes ranging from 0.3 to 1.6 ng/ml; data available on request).

#### 2.4.2 Indirect ELISA for quantification of antigen-specific titers

Clear 96-well plates were coated (1 hour at 37 °C or overnight at 4 °C) with 1 μg KLH or 0.5 μg diphtheria toxin in 100 μl PBS. The plates were washed and blocked and washed again. Serum and plasma samples were analyzed in duplicate. An antigen-specific positive serum sample was serially diluted in PBS (0.1% BSA) to form a linear standard curve. Following incubation (1 hour, 37 °C) the plates were washed and secondary antiserum (rabbit anti-chicken IgY:HRP, diluted 1:10,000 in PBS, 0.1% BSA) was added. Following incubation (1 hour at 37 °C) the plates were washed and 50 μl/well TMB was added. After 10–15 minutes the reaction was terminated by adding 100 μl/well 1 M H_2_SO_4_ and the absorbances were measured at 450 nm.

### 2.5 Preparation of splenocytes

Dissected spleens were trimmed from fat and connective tissue and single cell suspensions were prepared by forcing the passage of splenic tissue through a fine metallic mesh back into fresh cell culture medium (RPMI 1640 with GlutaMAX; Thermo Fisher Scientific) with 10% heat inactivated fetal bovine serum (Sigma Aldrich), 100 U/ml penicillin and 100 μg/ml streptomycin (Sigma Aldrich). Cell clusters were disrupted by pipetting and the neat suspension was transferred to new tubes. The cells were washed by decanting the supernatant after centrifugation (10 minutes, 200 g), and subsequent resuspension in fresh medium. Splenocytes were isolated from the cell suspension by density gradient centrifugation with Lymphoprep™ (Stemcell Technologies, Vancouver BC, Canada) according to the manufacturer’s instructions. The isolated cells were washed again, counted in a hemacytometer, and used for analysis immediately.

### 2.6 ELISPOT for splenic plasma cells

To quantitate antigen-specific splenic plasma cells an ELISPOT was utilized, similar to what has been utilized in chickens in the past [36]. Mixed cellulose ester membrane plates (MAHAS45010; Merck Millipore) were coated overnight at room temperature with 1 μg goat anti-chicken IgY or 0.5 μg KLH in 100 μl PBS pH 7.4. The plates were washed in PBS and 200 μl/well cell medium was added. Following incubation for 2 hours, the plates were washed in PBS and splenocytes (< 500,000 cells/well diluted in fresh cell medium) were added to the wells in triplicate and plates were incubated for > 32 h in an incubator (40 °C and 5% CO_2_). The plates were washed in PBS, 0.05% Tween 20 and incubated with 100 μl/well rabbit anti-chicken IgY:HRP diluted 1:1000 in PBS (1% BSA) for 2 hours at 40 °C and 5% CO_2_. The plates were washed in PBS, 0.05% Tween 20, and subsequently PBS without Tween, before 100 μl/well 4-Chloro-1-Naphthol (Sigma Aldrich; cat. no. C8302) was added. The plates were incubated for 45–60 minutes and the reaction was stopped by thorough rinsing of the plates in Milli-Q water. Spots were counted on the dried plates using an automated plate reader (CTL-ImmunoSpot S6; CTL, Shaker Heights OH, USA). Numbers of plasma cells producing α-KLH IgY was related to the total number of IgY-producing plasma cells in the same sample.

### 2.7 Data treatment

The concentration of α-KLH IgY in samples was quantified in relation to an aliquoted standard sample with a concentration defined as 1000 AU (arbitrary units)/ml. To improve the ability to distinguish low levels of specific antibodies from unspecific binding in the assay, samples were further normalized by factoring in the sample total IgY concentration. To approximate a normal distribution, data on circulating IgY concentrations and the normalized antigenic responses were log-transformed prior to all analyses. A measurable antigenic response was defined as a response exceeding the average of the pooled negative controls (non-immunized hens) by two standard deviations. An immunogen dose was considered eliciting a positive antigenic response only when all the individuals receiving it produced a positive antigenic response. Naïve serum samples were tested for initial differences using independent-samples t-tests adjusted for unequal variances. Antigenic responses and total circulating levels of IgY were tested separately. Inter-breed differences in immune response were tested for using a repeated measures ANOVA design with breed and immunogen dose (as a covariate) offered as predictors; degrees of freedom were adjusted using Greenhouse-Geisser correction as error covariances did not meet the requirement of sphericity. ANOVAs were also used to test whether increasing complexity of the co-administered protein matrix had a suppressive effect on the immune response toward the model immunogens, by leaving out the individuals that received no competing protein mix and offering group (the protein matrix used) and immunogen dose as predictors. To allow for detection of small populations of α-KLH plasma cells in the ELISPOT assay, wells were loaded with very high numbers of splenocytes. This can potentially inflate the number of false positives. Thus, to better establish a negative range when analyzing the data, the number of α-KLH IgY producing splenocytes associated with detectable circulating levels of α-KLH IgY was used. A positive response was defined as a number of α-KLH IgY positive splenocytes exceeding the average of the negatives by two standard deviations.

## 3 Results

### 3.1 Protein mixtures

**Fig. 1:**
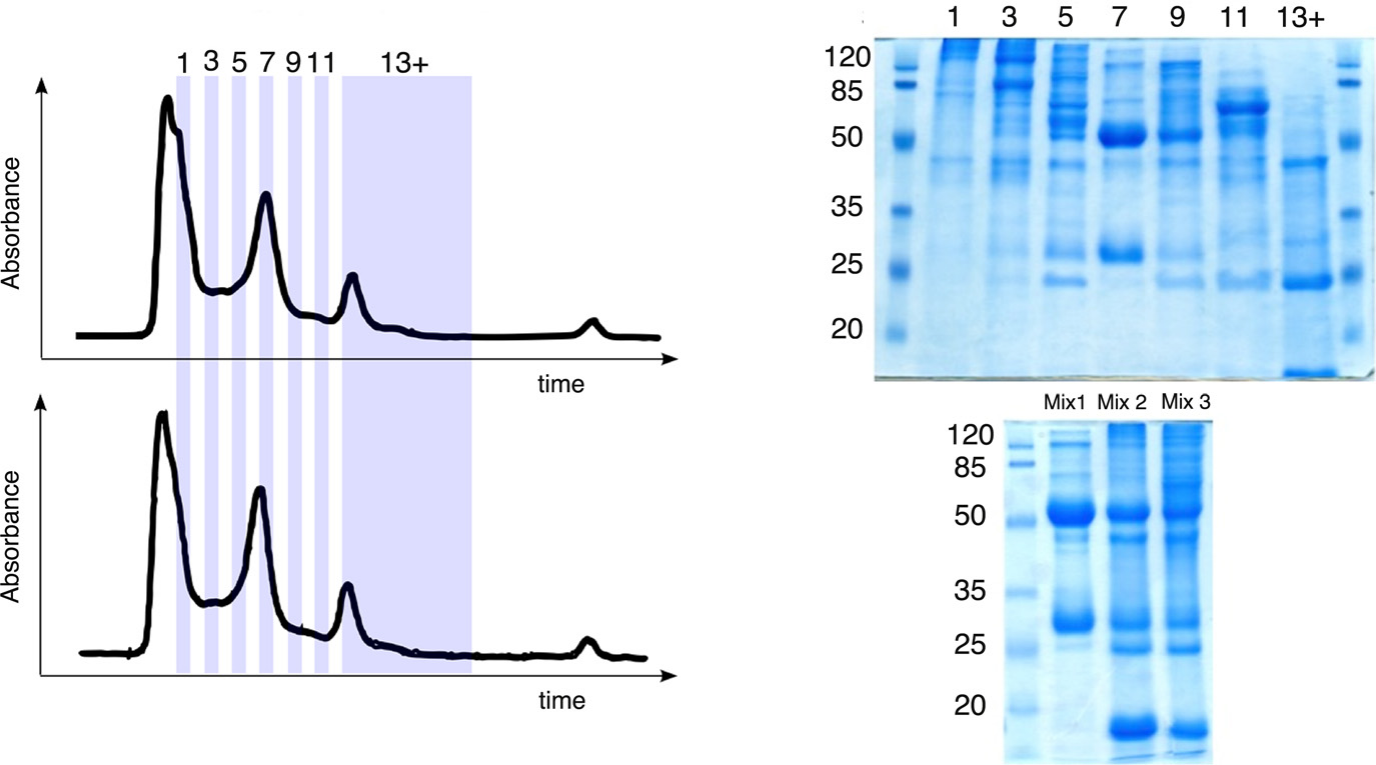
Composition and construction of protein mixes. Left: For an overview of the size-exclusion chromatography, two UV (λ: 280 nm) absorption chromatograms (arbitrarily selected from 9 batch runs) have been digitally traced from a paper source, been aligned, and the fractions used in constructing the protein mixes have been defined. Top right: Reducing SDS-PAGE analysis of placental protein fractions separated by size exclusion chromatography (15 μg loaded total protein per lane). Molecular weight markers (numbers indicate size in kDa) have been included on both sides of the gel. Bottom right: The three protein mixtures – post combination – analyzed by reducing SDS-PAGE (50 μg total protein per lane).

The water-soluble placental proteins were separated using size exclusion chromatography and a total of 13 fractions were pooled from nine batch-wise fractionations: Fractions 1, 3, 5, 7, 9, 11, and a pool collecting all fractions from 13 and onward, were used to prepare Mix 1, 2, and 3 (Fig. 1). Mix 1 consisted of a single fraction (7), Mix 2 consisted of three combined fractions (1, 7, and the final fractions), and Mix 3 consisted of all the listed fractions.

### 3.2 Experiment 1: comparing the antigenic response in two chicken breeds

**Fig. 2:**
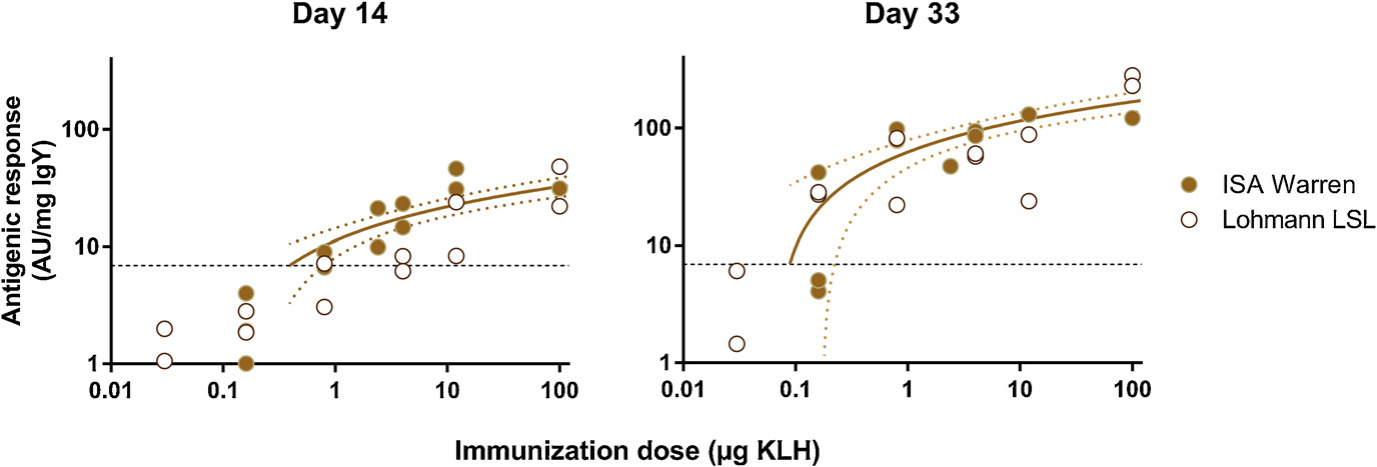
Antigenic response toward KLH for ISA Warren and Lohmann LSL hens. Results are shown for Day 14, after the primary immunization, and Day 33, after two booster immunizations (results for Day 28 are not shown). The two breeds did not appear to differ significantly in their antigenic response to the immunogen (KLH); combining data from all birds, trends have been plotted (best-fit semi-log interpolation lines with 95% CI, defined for responses exceeding the detection limit) outlining the dose-response relationship. The (lower) detection limit for a positive response is indicated by the dashed line.

Both breeds of hen had a clear antigenic response toward immunizations with KLH, with doses ≥ 0.8 μg KLH ensuring a measurable response – largely comparable to the response against doses of 100 μg – in both breeds following three successive immunizations (Fig. 2). Serum from naïve ISA Warren hens (obtained on day 0, before immunizations) displayed higher levels of unspecific binding than did serum from Lohmann LSL hens (T-test: t_21_ = −3.7, p < 0.001). By contrast, the Lohmann LSL hens responded to the booster immunizations by producing higher levels of circulating IgY (the effect of the interaction time × breed on total IgY concentrations: F_1.7,38_ = 21, p < 0.001). These differences in dynamics of the antigenic response canceled out over time, however, with the antigenic response toward KLH not differing significantly between the two breeds (F_1,22_ = 0.001, p = 0.97).

### 3.3 Experiment 2: titration of KLH and diphtheria toxin against a mixture of competing antigens.

**Fig. 3:**
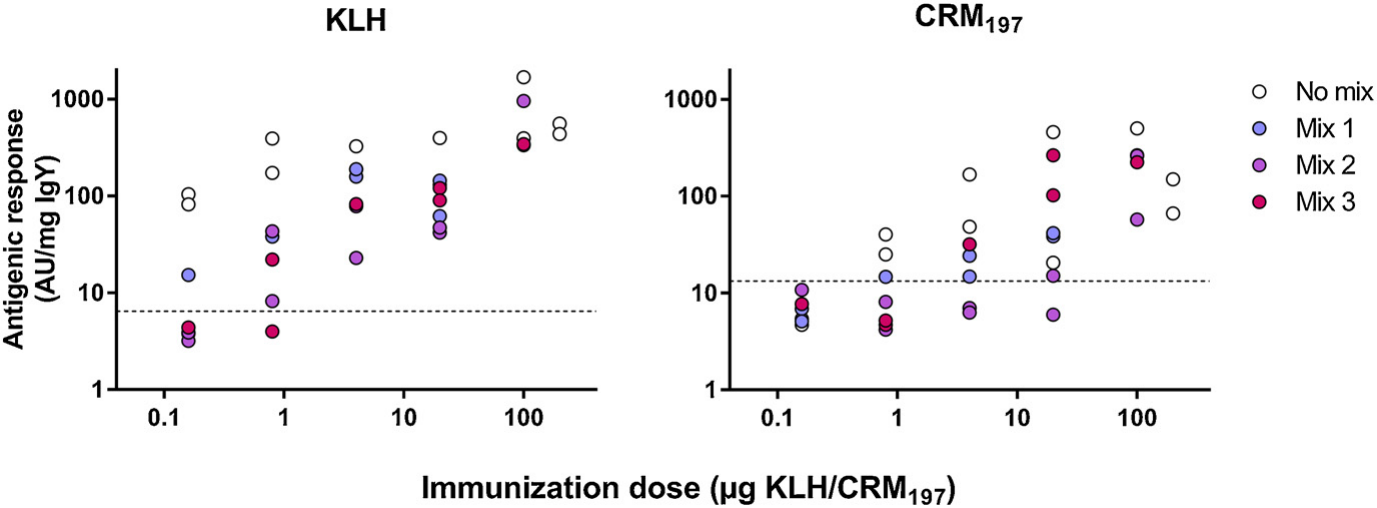
Antigenic response toward KLH and diphtheria toxin (CRM_197_) on day 39. The immunogens were provided either free of competing proteins (No mix) or in combination with proteins mixes of increasing complexity (Mix 1–3, numbered in order of increasing complexity). An interfering effect can be seen where co-immunizations with mixes reduce the antigenic response to the two model immunogens. It is however not possible to discern a clear relationship in the extent of this interference between the mixes of different complexity. The detection limits for a positive response are indicated by the dashed lines.

Of the two simultaneously administered model immunogens KLH was the more potent, eliciting a measurable antigenic response at doses ≥ 0.16 μg (as also found for Lohmann LSL in Exp. 1). By contrast, doses ≥ 0.8 μg had to be utilized to ensure a response toward the diphtheria toxin. The antigenic response of both model immunogens could be suppressed through the addition of protein mixtures of differing complexity. The antigenic response could subsequently be rescued by increasing the doses of the immunogens: KLH elicited a measurable response at doses ≥ 4 μg whereas diphtheria toxin had to be supplied in doses ≥ 100 μg in order for the antigenic response to reliably exceed background levels (Fig. 3). The suppressive effect of the different matrices appeared to be independent of the composition of the mixtures (Effect of mix on antigenic response at day 39: KLH, F_2,20_ = 1.2, p = 0.31; CRM_197_, F_2,19_ = 3.1, p = 0.07) and relative abundance of various placental proteins.

### 3.4 Experiment 3: titration of competing antigens against a constant dose of KLH

**Fig. 4:**
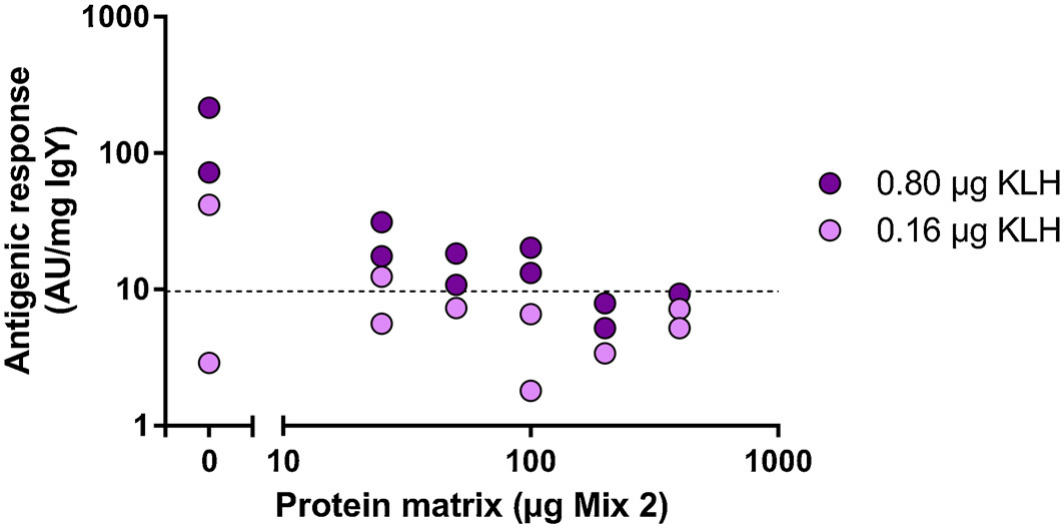
Suppression of the antigenic response toward KLH (either 0.16 or 0.80 μg) by simultaneous immunization with variable concentrations of a protein matrix (Mix 2 from Exp. 2). The detection limit for a positive response is indicated by the dashed line.

Unlike our findings in the previous experiment, immunizations with 0.16 μg KLH were insufficient to ensure a measurable antibody response in Lohmann LSL hens (Fig. 4). Simultaneous immunization with 0.8 μg KLH and different amounts of competing antigens resulted in a significant reduction of the KLH-specific response which was fully suppressed in the presence of ≥200 μg placental protein mixture.

**Fig. 5:**
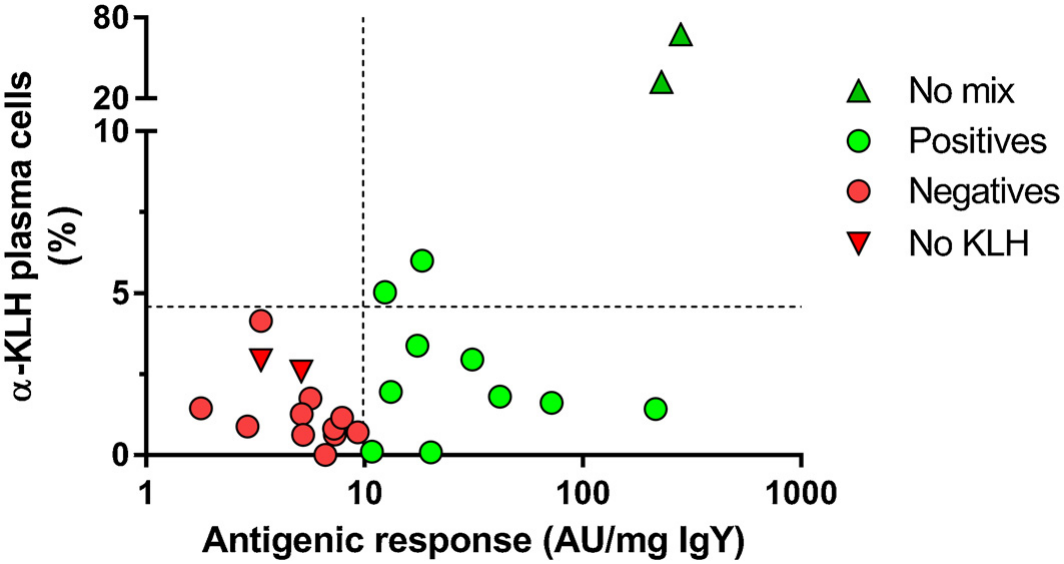
Number of plasma cells producing antibodies toward KLH, as a percentage of the total population of IgY-producing splenocytes, graphed as a function of the corresponding antigenic response. Individuals immunized with 100 μg KLH, and no competing protein matrix, (No mix; from Exp. 1) were included as a positive reference. The magnitude of a positive response to the immunizations can in no way be predicted by the number of splenocytes participating in it, and in most cases not even be discerned from a negative response. The detection limits for a positive response are indicated by the dashed lines.

Following three successive immunizations with 100 μg KLH, the population of IgY-producing plasma cells specific for KLH constituted up to 70% of the total number of IgY-producing plasma cells in the spleens of the immunized chickens. Three successive immunizations with 0.8 μg KLH was capable of inducing an antibody response equivalent in magnitude to 100 μg KLH. However, when analyzing the number of α-KLH plasma cells in an ELISPOT, individuals with a positive antigenic response toward 0.8 μg doses of KLH could not be distinguished from individuals testing negative. This was also the case for α-KLH positive individuals, who had received a competing protein matrix (Fig. 5).

## 4 Discussion

In trying to predict the number of simultaneous immunizations that can be used successfully in a chicken model, we tried to characterize the mechanisms limiting the number of simultaneous antigenic responses. We hypothesized that the number of splenic plasma cells would be relatable to the antigenic response they were a part of. The limited resources of the spleen coupled with a critical number of plasma cells needed for a measurable immune response toward an immunogen could then be used to estimate the number of antigens it could be administered in the presence of. The fact that two antigenic responses of comparable magnitude could be produced by wildly disparate numbers of plasma cells precluded this approach however. Whereas it has previously been shown that plasma cell populations and resulting circulating concentrations of IgG are not directly correlated in humans [37,38], it was still quite surprising to find that the concentration of circulating α-KLH IgY could in no way be predicted from the splenic content of α-KLH IgY producing plasma cells. Circulating concentrations of specific antibodies usable for polyclonal antibody production, could be produced while recruiting 70% of the splenic plasma cells, but just as easily be associated with less than 5%. This difference can possibly be explained as a greater level of activation of the smaller population of plasma cells when the immune system is under greater strain. But it is also possible that the magnitude of the antigenic response is more directly related to populations of plasma cells disseminated to other tissues – as has been demonstrated in some studies [39] – such as bone marrow or mucosa-associated lymphoid tissue. Future studies into the antigenic response toward immunizations in chickens would do well to focus not only on the magnitude of the response, but also its localization.

For the present study, two potent model immunogens were used in order to avoid non-responder individuals and to have a clear (“benchmark”) antigenic response with which to work. This provides a “best case scenario” with respect to estimating the capacity of a bird’s adaptive immune response: Lohmann LSL hens would, for example, respond reliably to doses of KLH as low as 0.8 μg. When co-administering the immunogens with a protein matrix, the response to low doses could be suppressed. To rescue the response, the immunogen dose had to be increased by more than an order of magnitude. We could not find a suppressing effect of increasing the complexity of the matrix of competing proteins, but only from the absolute concentration of proteins. Whereas we cannot rule out our not having reduced the complexity enough to see an effect – even the least complex mix contained a high number of different proteins – it appears absolute concentrations were more important for the suppressive effect.

It has been suggested that no more than 1 mg of protein material can, or for animal welfare reasons should, be used in immunizing a hen [2,4]. With both KLH and CRM_197_ producing high antigenic responses when administered in doses below 5 μg our initial hypothesis seemed to hold: pushing the limits slightly – using multiple deposition sites to distribute a somewhat higher total volume – a single individual could be immunized with thousands of immunogens at these doses. We would be disappointed however: When co-administering the model immunogens with a matrix of competing proteins we found that more than ten times higher concentrations of both immunogens were needed to elicit a measurable antigenic response. This greatly limits the number of proteins that can be used in a multiplexed immunization. Although there will quite often be proteins/peptides that the immune system will overlook, even a potent immunogen like CRM_197_ can be completely overshadowed when co-administered with 400 μg of competing proteins. With the tested model immunogens requiring doses of 4 μg (KLH) and 100 μg (CRM_197_) for a reliable response when multiplexed, the maximum number of immunogens that can be provided at once shrinks significantly, suggesting a number between 10 and 100. With the two proteins having proven to be extremely immunogenic, it is likely that a true number of (less potent) immunogens that a hen can elicit a usable − in the context of producing polyclonal antibodies – immune response toward in a multiplexed immunization is barely in the double digits. These findings agree well with a previous study in mice by De Masi and collaborators who reported an average no-response rate of one-in-four when using as few as ten immunogens [20].

Although we know that the immune system can produce immunoglobulins toward scores of targets (whether defined as whole proteins or epitopes) at the same time, only a few targets will give rise to immunoglobulin responses high enough for it to make sense to isolate and purify them for analytical/diagnostic use. It is furthermore hard to predict which epitopes will present the lucky few targets. When utilizing multiplexed immunizations, increasing the number of immunogens beyond a certain threshold is pointless. As the number of targets grows, so too does the relative fraction of immunogens that do not elicit a usable immune response: any gains that could be had from multiplexing the immunizations effectively being negated by the increasing probability of the subjects not responding.

The suitability of two different chicken breeds for antibody production was also analyzed in the present study. Here, we were able to find only limited differences in the immune response between Lohmann LSL and ISA Warren. Similarly to a previous study [40], the differences we found were so slight that we could find no reason to advocate one breed over the other for antibody production.

In conclusion, the present findings confirm that the hen’s immune system can be directed to producing multiple antisera at the same time. For proteins such as KLH, only minute concentrations need to be used to ensure circulating IgY concentrations usable for isolating polyclonal serum when immunized alone. When co-administered with other proteins, higher concentrations are needed to ensure a predictable response. This greatly limits the number of immunogens that can be multiplexed. At present we would recommend a cautious approach when utilizing more than ten immunogens at once and we believe that more than a hundred is a fruitless endeavor.

## Declarations

### Author contribution statement

Tina M. Kousted, Otto Kalliokoski: Conceived and designed the experiments; Performed the experiments; Analyzed and interpreted the data; Wrote the paper.

Sofie K. Christensen: Performed the experiments; Analyzed and interpreted the data; Wrote the paper.

Jakob R. Winther, Jann Hau: Conceived and designed the experiments; Analyzed and interpreted the data; Wrote the paper.

### Competing interest statement

The authors declare no conflict of interest.

### Funding statement

This work was supported by a generous grant from the Innovation Fund Denmark (project no. 88-2013-1).

### Additional information

No additional information is available for this paper.

## Figures and Tables

**Table 1:** Design for Experiment 1. Number of hens (n) immunized with a given dose of KLH (in μg). The uneven subject distribution arose from a miscommunication and practical constraints, but should have no effect on the overall hypothesis tests.

KLH (μg)	ISA Warren (n)	Lohmann LSL (n)
0	2	2
0.030	–	2
0.16	3	2
0.80	2	2
2.4	2	–
4.0	2	2
12	2	2
100	1	2

**Table 2:** Design for Experiment 2. Number of hens (n) immunized with a given dose of model immunogens (KLH and diphtheria toxin CRM_197_) and a mix of competing proteins (numbered by increasing complexity from 1–3).

KLH (μg)	CRM_197_ (μg)	Mix 1 (μg)	Mix 2 (μg)	Mix 3 (μg)	Lohmann LSL (n)
0	0				2
0.16	0.16				2
0.80	0.80				2
4.0	4.0				2
20	20				2
100	100				2
200	200				2
0	0	400			2
0.16	0.16	400			2
0.80	0.80	400			2
4.0	4.0	390			2
20	20	360			2
100	100	200			2
0	0		400		2
0.16	0.16		400		2
0.80	0.80		400		2
4.0	4.0		390		2
20	20		360		2
100	100		200		2
0	0			400	2
0.16	0.16			400	2
0.80	0.80			400	2
4.0	4.0			390	2
20	20			360	2
100	100			200	2

**Table 3:** Design for Experiment 3. Number of hens (n) immunized with a given dose of KLH and a mix of competing proteins (Mix 2 from Exp. 2).

KLH (μg)	Mix (μg)	Lohmann LSL (n)
0	0	2
0.16	0	2
0.16	25	2
0.16	50	2
0.16	100	2
0.16	200	2
0.16	400	2
0.80	0	2
0.80	25	2
0.80	50	2
0.80	100	2
0.80	200	2
0.80	400	2
